# Supplementary material for: Electric Fields Regulate In Vitro Surface Phosphatidylserine Exposure of Cancer Cells via a Calcium-Dependent Pathway
Source: Biomedicines. 2023 Feb 6;11(2):466. doi: 10.3390/biomedicines11020466 (PMC9953458; doi:10.3390/biomedicines11020466)
Supplement: Supplementary file 1 [file biomedicines-11-00466-s001.zip › biomedicines-1991769-supplementary.pdf]

**Supplemental Materials**

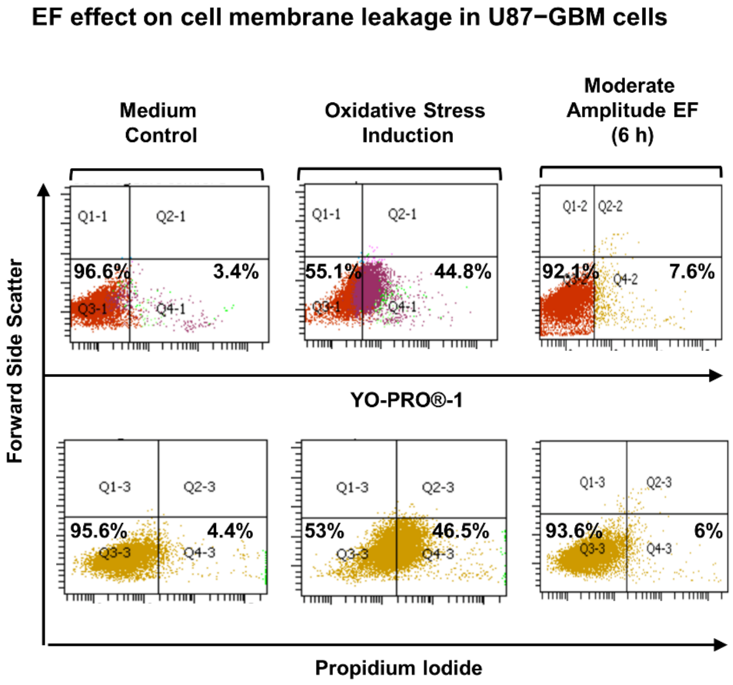


**Figure S1:** Moderate amplitude electric field does not disturb the cell membrane integrity. Flow cytometric measurements of YO-PRO-1 and Propidium Iodide demonstrate no change in cell staining in U87-GBM cancer cells following moderate amplitude EF for 6 h (top & bottom right) compared to medium control (top & bottom left panel).

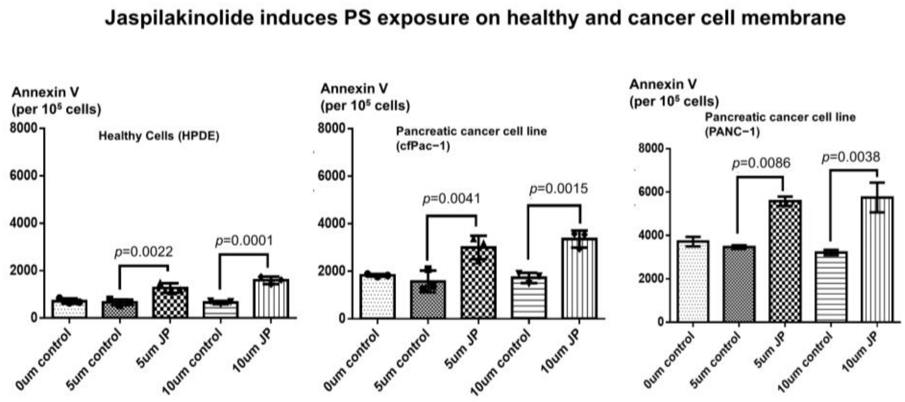


**Figure S2:** Actin polymerization remodeling by Jasplakinolide and electric field in cancer cells is associated with increased PS externalization. Flow cytometric measurements of annexin V on indicated healthy cells (left panel) and cfPac-1(middle panel) and PANC-1 (right panel) pancreatic cancer cell lines under treatment with Jaspilakinolide with indicated concentrations.

## Oxidative stress increases p-38 MAPK activation in cancer cells

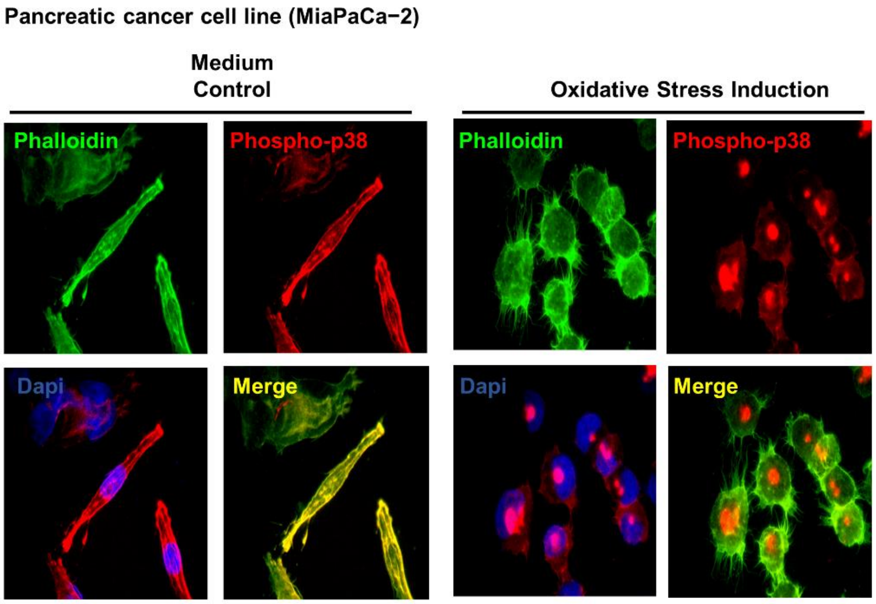


**Figure S3:** Oxidative stress activates P-38 MAPK in cancer cells. Immunofluorescence staining of MiaPaCa-2 pancreatic cancer cells cultured with DMEM for 24 hrs (left panel) and with 1.2 mM hydrogen peroxide for 20 min (right panel) shows that oxidative stress increases nucleus translocation of p-p38 MAPK, which indicates the activation of P-38 MAPK.
